# *RASSF1* tumor suppressor gene in pancreatic ductal adenocarcinoma: correlation of expression, chromosomal status and epigenetic changes

**DOI:** 10.1186/s12885-016-2048-0

**Published:** 2016-01-12

**Authors:** Eliana Amato, Stefano Barbi, Matteo Fassan, Claudio Luchini, Caterina Vicentini, Matteo Brunelli, Giuseppe Malleo, Aldo Scarpa, Giorgio Malpeli

**Affiliations:** ARC-NET Centre for Applied Research on Cancer, Department of Pathology and Diagnostics, The Hospital and University of Verona, Verona, Italy; Department of Surgery and Oncology, The Hospital and University of Verona, Verona, Italy; Department of Pathology, The Hospital and University of Verona, Verona, Italy

**Keywords:** Ductal adenocarcinoma, Pancreas, *RASSF1*, Rassf1a, Methylation, Pyrosequencing, Quantitative PCR

## Abstract

**Background:**

The Ras Association Domain Family Member 1 (*RASSF1*) is one of the most frequently reported methylation-inactivated tumor suppressor genes in primary pancreatic ductal adenocarcinomas (PDAC). Limited information is still available about the impact of *RASSF1* gene silencing on the expression of its different isoforms in neoplastic cells.

**Methods:**

A series of 96 primary PDAC, with known clinico-pathological parameters, was tested for *RASSF1* methylation status by methylation-specific PCR, *RASSF1* locus copy number alterations by fluorescence in situ hybridization, and Rassf1a protein expression by immunohistochemistry. A further series of 14 xenografted primary PDAC and 8 PDAC-derived cell lines were tested to obtain a detailed methylation mapping of CpG islands A and C of the *RASSF1* locus by pyrosequencing and to evaluate the expression of Rassf1 variants by qRT-PCR.

**Results:**

Methylation of CpG island A of the *RASSF1* gene was observed in 35 % of the tumors and allelic loss of *RASSF1* locus was seen in 30 disomic and in 20 polysomic cases (52 %). Rassf1a immunohistochemical expression was downregulated in half of primary PDAC, and this downregulation was neither correlated with methylation of *RASSF1* promoter nor with *RASSF1* copy number alterations. *RASSF1* status did not influence patients’ prognosis. The expression of the seven RASSF1 isoforms in xenografts and cell lines showed that RASSF1A, RASSF1B, and RASSF1C isoforms were present in all xenografts and cell lines, whereas RASSF1D, RASSF1E, and RASSF1F isoforms were variably expressed among samples. RASSF1G was never expressed in either xenografts or cell lines. The variable expression of RASSF1 isoforms in PDAC xenografts and cell lines was not dependent on *RASSF1* methylation status of CpG islands A and C.

**Conclusions:**

*RASSF1* alterations occurring in PDAC mainly consist in variations of expression of the different isoforms. Different genetic mechanisms seem to contribute to *RASSF1* deregulation in this setting, but *RASSF1* methylation does not seem to substantially affect RASSF1 isoforms expression.

**Electronic supplementary material:**

The online version of this article (doi:10.1186/s12885-016-2048-0) contains supplementary material, which is available to authorized users.

## Background

A recent genome-wide profiling of DNA methylation of 167 pancreatic ductal adenocarcinomas (PDAC) identified 3522 genes that showed an altered methylation status [1]. Among these genes, the Ras Association Domain Family Protein 1 (*RASSF1*) locus showed frequent hypermethylation, confirming a previous report of *RASSF1* locus hypermethylation in two thirds of primary PDAC and seven of eight PDAC cell lines [2].

*RASSF1* is a putative tumor suppressor gene that controls tumor growth by inhibiting the RAS pathway [3–6]. Recent studies assigned additional complex roles to *RASSF1* in cell life such as promotion of apoptotic signaling, microtubule stabilization and mitotic progression [7–12]. As constituent of RAS/PI3K/AKT, RAS/RAF/MEK/ERK and Hippo cancer pathways, an important role in solid tumors has been suggested [12–14] and, in this context, *RASSF1* is one of the most frequently reported methylation-inactivated tumor suppressor genes [12, 15]. It has also been reported a prognostic role for *RASSF1* methylation in a series of cancer types (liver, gastric, prostate, bladder, kidney, brain and pituitary neoplasms), where an altered methylation status has been associated to a more aggressive biological behaviour [13, 16–21]. *RASSF1* methylation has been also reported as a good predictor of response to gemcitabine-chemotherapy in lung cancer [22]. *RASSF1* locus on chromosome 3p21.3 generates seven transcript isoforms (RASSF1 A-G) by differential promoter usage and alternative splicing [23]. Two CpG islands are associated to *RASSF1*: 1) CpG island A, common to *RASSF1A*, *D*, *E*, *F* and *G,* in the promoter region, and 2) CpG island C in the regulatory region of *RASSF1B* and *C*. RASSF1A and RASSF1C are the major isoforms and they are ubiquitously expressed in normal tissues [23]. Expression of RASSF1 isoforms has never been studied in primary PDAC, whereas in PDAC cell lines RASSF1A was found to be expressed only in cells resulting unmethylated with a qualitative methylation specific PCR [2]. Among the remaining RASSF1 variants, only RASSF1F was tested and found frequently expressed in PDAC cell lines [2].

PDAC is characterized by a large number of chromosomal alterations among which loss of heterozygosity (LOH) at chromosomal arm 3p21.3, where RASSF1 resides, has been reported to occur as ranging from 43 to 75 % of cases [24–26].

In this study, we report the evaluation of 96 primary PDAC for Rassf1a protein immunohistochemical expression, *RASSF1* gene copy number by FISH, and methylation of *RASSF1* locus by methylation-specific PCR. To get insight into the correlation of expression data and methylation of *RASSF1*, we used 14 primary PDACs xenotransplanted in nude mice and 8 PDAC cell lines to perform a detailed analysis of methylation of CpG islands A and C of the *RASSF1* locus by DNA pyrosequencing and mRNA expression analysis of RASSF1 variants by quantitative RT-PCR.

## Methods

### PDAC tissues

A series of 96 formalin-fixed and paraffin-embedded (FFPE) primary PDAC, whose clinico-pathological characteristics are shown in Table 1, was considered. The series was studied for Rassf1a expression by immunohistochemistry (IHC) and *RASSF1* gene copy number by fluorescence *in situ* hybridization (FISH) by using 4 tissue microarrays (TMAs). The Manual Tissue Arrayer MTA-1 (Beecher Instruments, Silver Spring, MD) was used in the assembling of the TMAs and three 1 mm tissue cores *per* case were included. Ten additional normal pancreatic parenchyma cores were used as controls and integrated in the TMAs.

**Table 1:** Distribution of 96 pancreatic ductal adenocarcinomas grouped by *RASSF1/*CEP3 status and clinico-pathological features

Parameter	Class	RASSF1 retained	RASSF1 loss	RASSF1 loss	RASSF1 gain
		CEP3 diploid	CEP3 diploid	CEP3 polyploid	CEP3 polyploid
Sex	F	14 (56.0 %)	11 (36.7 %)	8 (40.0 %)	6 (28.6 %)
	M	11 (44.0 %)	19 (63.3 %)	12 (60.0 %)	15 (71.4 %)
Age	Mean	63	58	64	63
pT	T3	25 (100.0 %)	28 (93.3 %)	17 (85.0 %)	20 (95.2 %)
	T4	0 (0.0 %)	2 (6.7 %)	3 (15.0 %)	1 (4.8 %)
pN	N0	5 (20.0 %)	7 (23.3 %)	4 (20.0 %)	3 (14.3 %)
	N1	20 (80.0 %)	23 (76.7 %)	16 (80.0 %)	18 (85.7 %)
pM	M0	24 (96.0 %)	29 (96.7 %)	18 (90.0 %)	18 (85.7 %)
	M1	1 (4.0 %)	1 (3.3 %)	2 (10.0 %)	3 (14.3 %)
Grading	G1	0	1 (3.3 %)	0	0
	G2	14 (56.0 %)	17 (56.7 %)	11 (55.0 %)	12 (57.1 %)
	G3	11 (40.0 %)	12 (40.0 %)	9 (45.0 %)	9 (42.9 %)
TNM	II	5 (20.0 %)	7 (23.3 %)	3 (15.0 %)	3 (14.3 %)
	III	19 (76.0 %)	20 (66.6 %)	12 (60.0 %)	15 (71.4 %)
	IV	1 (4.0 %)	3 (10.0 %)	5 (25.0 %)	3 (14.3 %)
TOTAL	-	25	30	20	21

### PDAC xenografts and cell lines

Fourteen fresh primary PDAC xenografted in nu/nu mice (mean age 57 ± 7; Male/Female = 7/7; T3N0M0 = 6, T3N1M0 = 8; G2 = 10, G3 = 4) [27] and 8 PDAC-derived cell lines (PACA3, PACA44, PT45, CFPAC, PC, HPAF, PSN, PANC1) [28] were studied for DNA methylation at *RASSF1* locus and mRNA expression of RASSF1 isoforms. Frozen material from neither primary tumors nor their normal counterparts for these xenografted cancers was available. Cell lines were grown in RPMI 1640 supplemented with 2 mM glutamine and 10 % FBS and incubated at standard conditions (37 °C, 5 % CO_2_).

### Ethics

The materials used have been collected under Program 853 protocol 298CE 15/02/2002 and Program 1885 protocol 52438 on 23/11/2010. The protocols include informed consent of the patient and were approved by the local ethics committee of the Integrated University Hospital Trust of Verona. The first approval (prog. 853) regarded the collection of pancreas samples for use in molecular research studies. This was later updated (prog. 1885) for the creation of a coordinated biobank for the collection of samples from all cancer patients that included neoplastic and associated local and distant normal tissue. The approved programs include tissue processing and storage methods of FFPE tissues of both neoplastic and normal tissue, including the creation of tissue microarrays. The latter program included amendments to address the later regulatory issues of data disclosure in genomic studies. Animal housing and all the protocols involving the use of experimental animals in this study were carried out with the authorization of the Italian Ministry of Health (approved protocol N. 184/2008-B).

### Immunohistochemistry (IHC)

Immunostaining was performed on 4 μm-thick FFPE sections using anti-Rassf1 antibody (HPA040735; Atlas antibodies, Stockholm, Sweden;1:200 dilution) with an automatic stainer (Bond instrument, Vision Biosystem Leica, Milan, Italy) as described previously [29, 30]. Appropriate positive and negative controls were run concurrently. Rassf1 immunostaining was considered positive when cells showed unambiguous cytoplasm staining. Sparse normal exocrine cells showed nuclear staining, which was not retained for scoring. The intensity of cytoplasmic staining was scored in a four-tiered scale: 0 = negative, 1 + = weak staining, 2 + = moderate staining, 3 + = strong staining, similarly to other immunohistochemical study [31].

### Fluorescence in situ hybridization (FISH)

FISH assay for *RASSF1* locus was performed on four 4-μm-thick sections from TMAs, using a home-made Spectrum Orange labeled DNA probe for the relevant region and a commercially available conjugated Cy3, Spectrum Green labeled, centromeric enumeration probe (CEP3) (Abbott-Vysis, Downer Grove, IL, USA). Briefly, BAC clone specific for chromosome 3p (RP11-894C9), mapping at 3p21.31 and belonging to the Roswell Park Cancer Institute libraries (Peter J. de Jong at http://bacpac.chori.org/) was selected. Paraffin sections were hybridized with the probe labeled by nick translation [32], using 500 ng of probe labeled with Fluorolink Cy3-dUTP or Fluor-X-dCTP (Amersham, Buckinghamshire, UK). For each sample, at least 110 nuclei were counted for *RASSF1* signals and alpha-satellite sequences of chromosome 3 centromere (CEP3), as previously reported [33]. Results were expressed as the mean value of *RASSF1* copy number: CEP3 signal ratio per cell.

### Nucleic acids extraction

Nucleic acids were prepared from FFPE primary PDAC tissues, and from frozen sections of PDAC xenografts. Tumor cellularity was manually enriched by microdissection to at least 70 %. DNA was purified by QiAamp DNA Mini Kit (Qiagen), following manufacturer’s instructions. RNA was extracted from PDAC cell lines and xenografts with TRIzol Reagent (Invitrogen) following manufacturer’s instructions, then treated with DNAse I (Invitrogen) for 15 min at room temperature, and finally incubated at 65 °C for 10 min for enzyme inactivation.

### Bisulfite treatment of DNA

DNA was chemically modified with sodium bisulfite using MethylSeq Kit (Applied Biosystems) to convert unmethylated cytosine to uracil, while methylated cytosines resist to conversion. DNA was incubated with sodium bisulfite for 16 h at 50 °C in a heat-block and used for subsequent experimental procedures.

### RASSF1A methylation-specific PCR (MSP)

Methylation-specific PCR (MSP) was performed according to Pizzi et al. [34]. Reference unmethylated DNA was from healthy donor peripheral blood mononuclear cells. Reference full methylated DNA was CpGenome Universal Methylated DNA (Chemicon International).

### Methylation-specific DNA pyrosequencing analysis

Bisulfite-modified DNAs were evaluated by pyrosequencing [35], using primers and conditions previously described [36]. DNA pyrosequencing provides the methylation status of single CpGs at the specific genomic region analyzed. The degree of methylation at each CpG position was determined from the ratio of C and T by the Pyro Q-CpG Software (Biotage AB). Pyrosequencing was performed on the sense and antisense strand of *RASSF1A* (nucleotides −163 to +262 in chromosome 3: 50353109–50353534, NC_0000003.10) and of *RASSF1C* (nucleotides −86 to +193 in chromosome 3: 50349706–50349985, NC_0000003.10).

### Identification of alternatively spliced mRNA isoforms of RASSF1 transcribed from CpG island A

RASSF1A is the main transcript isoform arising from the promoter containing CpG island A and the D, E, F and G isoforms originate from alternative splicing of RASSF1A. Taking advantage of their different length, isoforms A, D, E, F were identified by microfluidic chip-electrophoretic separation (DNA 1000 chip, 2100 Bioanalyzer, Agilent technologies) of PCR products. PCR amplification of cDNAs used primers designed in first and last exon of the gene (NCBI Reference Sequence NM_007182) and was performed as previously described [36]. As RASSF1G lacks the last exon of RASSF1, it was amplified separately with appropriate primers [36].

### Quantitative reverse transcription-polymerase chain reaction (qRT-PCR)

Messenger RNA expression of the major RASSF1 variants RASSF1A, RASSF1B and RASSF1C was determined in 14 xenografted PDAC and 8 PDAC cell lines. RNA samples were retrotranscribed to cDNA using the First Strand cDNA Synthesis Kit (Roche). A reverse transcriptase minus cDNA was prepared for each sample as a control. QRT-PCR was carried out as previously described [36] in 25 μl total volume containing 4 ng of cDNA, 1x Power SYBR Green I Master Mix (Applied Biosystems), 400 nM of each primer. After a starting denaturation for 10 min at 95 °C, 45 PCR cycles (15 s 95 °C and 1 min 60 °C) have been performed on ABI PRISM 7900HT SDS instrument (Applied Biosystems). The relative expression level was calculated using transcript level of *RPLPO* as reference gene and the standard (=1) was the average of the levels of expression of all samples. QRT-PCR data analysis was performed according to the comparative method following the User Bulletin #2 (Applied Biosystems).

### DNA sanger sequencing

*KRAS* (exons 2, 3, and 4) and *BRAF* (exon 15) genes mutational status was assessed by Sanger sequencing, as described elsewhere [37]. PCR products were purified using Agencourt AMPure XP magnetic beads (Beckman Coulter) and labelled with BigDye® Terminator v3.1 (Applied Biosystems). Agencourt CleanSEQ magnetic beads (Beckman Coulter) were used for post-labeling DNA fragment purification, and sequence analysis was performed on the Applied Biosystems 3130xl Genetic Analyzer.

### Statistical analysis

Pearson’s correlation (r) and Student’s *t-test* were used to compare mRNA expression and differentially methylated regions between groups of samples. Fisher’s exact test and Cox multivariate model were used to calculate correlations between results and clinico-pathological features. *p*-values less than 0.05 were considered statistically significant. Where applicable, the tests were two-tailed. For all the calculations, the R statistical software package was used (http://www.r-project.org).

## Results and discussion

### Rassf1a immunohistochemical expression is downregulated in half of primary PDAC

**Fig. 1:**
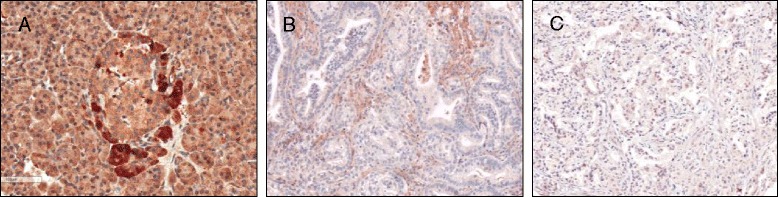
IHC Rassf1a expression in normal pancreas and PDAC. **a** Exocrine and islet cells consistently showed a moderate/strong (2+/3+) Rassf1a cytoplasmic immunoreaction. **b** PDAC with only few positive cells, and with a weak reactivity (1+); between neoplastic cells there is a nonspecific staining deposition. **c** One of the PDAC cases totally negative (0). Original magnifications 10x and 20x

Rassf1a expression was evaluated by IHC in 10 normal pancreas, 96 primary PDAC tissues, 14 PDAC xenografts and 8 PDAC cell line. Normal pancreas had clear Rassf1a 2+ or 3+ immunoreactivity (Fig. 1) that was uniform in acinar cells and insulae, although the latter showed some degree of heterogeneity from 2+ to 3+. Some cells at the periphery of the insulae, possibly corresponding to non-ß insular cells, were more strongly stained. PDAC tissues showed a uniform immunostaining among the three different cores of TMAs representative of each single PDAC. In summary, primary PDAC showed a variable expression from 0 to 3+ (Fig. 1), where 7 (7.3 %) cases were negative, 41 (42.7 %) were 1+, 35 (36.5 %) were 2+, and 13 (13.5 %) were 3+ (Additional file 1: Table S1). On the whole, 50 % of primary PDAC (48/96) showed a down-regulation (0/1+) of Rassf1a in comparison to normal pancreas (*p* = 0.0018, Fisher’s exact test). Rassf1a expression was not associated to any specific clinico-pathological parameter.

### Rassf1a immunohistochemical expression is downregulated in half of xenografted PDAC and PDAC cell lines

In xenografted PDAC, Rassf1a expression was 0 in 3 (21 %) cases, 1+ in 5 (36 %) cases and 2+ in 6 (43 %) cases (Additional file 1: Table S2). Rassf1a was thus down-regulated (0/1+) in 8 of 14 (57 %) xenografts. All PDAC cell lines expressed Rassf1a: 4 (50 %) had 1+, 3 (37.5 %) had 2+ and 1 (12.5 %) had 3+ (Additional file 1: Table S3). Rassf1a was thus down-regulated in 4 of 8 (50 %) PDAC cell lines.

### RASSF1 locus shows frequent copy number alterations in PDAC

**Fig. 2:**
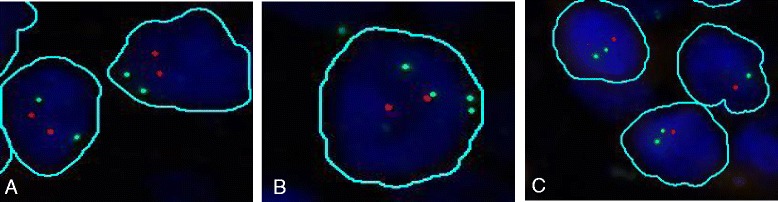
Representative FISH digitalized images of the *RASSF1* and chromosome 3 status in PDAC. The DNA probes used were a home-made Spectrum Orange for the locus specific *RASSF1* gene and a commercially available Spectrum Green enumeration *CEP3* probe: **a** diploid status for chromosome 3 and *RASSF1* loci; **b** gains of chromosome 3 (four signals) and two *RASSF1* signals reflecting loss in a chromosome 3 tetraploid nucleus; **c** loss of RASSF1 locus in two diploid chromosome 3 nuclei. The digitalized images are obtained by the High Technology Scan D-Sight/Fluo software (Visia Imaging, San Giovanni Valdarno, Italy), which also recognizes and circles individual neoplastic DAPI stained nuclei

According to CEP3 FISH data, chromosome 3 was in a disomic status in 55 PDAC and polysomic in 41 (from 3 to 7 signals, mean 3.7). *RASSF1* locus was retained in 46 cases and lost in 50 cases (Fig. 2). Thus, the combination of chromosome 3 ploidy status as assessed by CEP3 and *RASSF1* gene copy number generated four distinct classes: 1) *RASSF1* retained/*CEP3* diploid (25 cases), 2) *RASSF1* loss/*CEP3* diploid (30 cases), 3) *RASSF1* loss/*CEP3* polyploid (20 cases), and 4) *RASSF1* retained/*CEP3* polyploid (21 cases) (Table 1 and Additional file 1: Table S1). Cases distributed uniformly among the four classes; no significant association was observed considering *RASSF1* status and clinico pathological parameters. The presence of polysomy in *CEP3*, independently of the *RASSF1* status, was significantly associated to a worse prognosis (Additional file 2: Figure S1). Patients with both disomic *CEP3* and *RASSF1* had a similar prognosis compared to patients with disomic *CEP3* and loss of *RASSF1*.

### CpG island A of RASSF1A is frequently methylated in PDAC

Methylation-specific PCR (MSP) detected methylation of *RASSF1A* in 34 of 96 (35.4 %) PDAC (Additional file 1: Table S1). In particular, methylation was detected in 18 of 50 (36 %) PDAC with loss of *RASSF1* and in 16 of 46 (34.7 %) PDAC without loss. There was no correlation between methylation of CpG island A as assessed by MSP and IHC expression of Rassf1a.

**Fig. 3:**
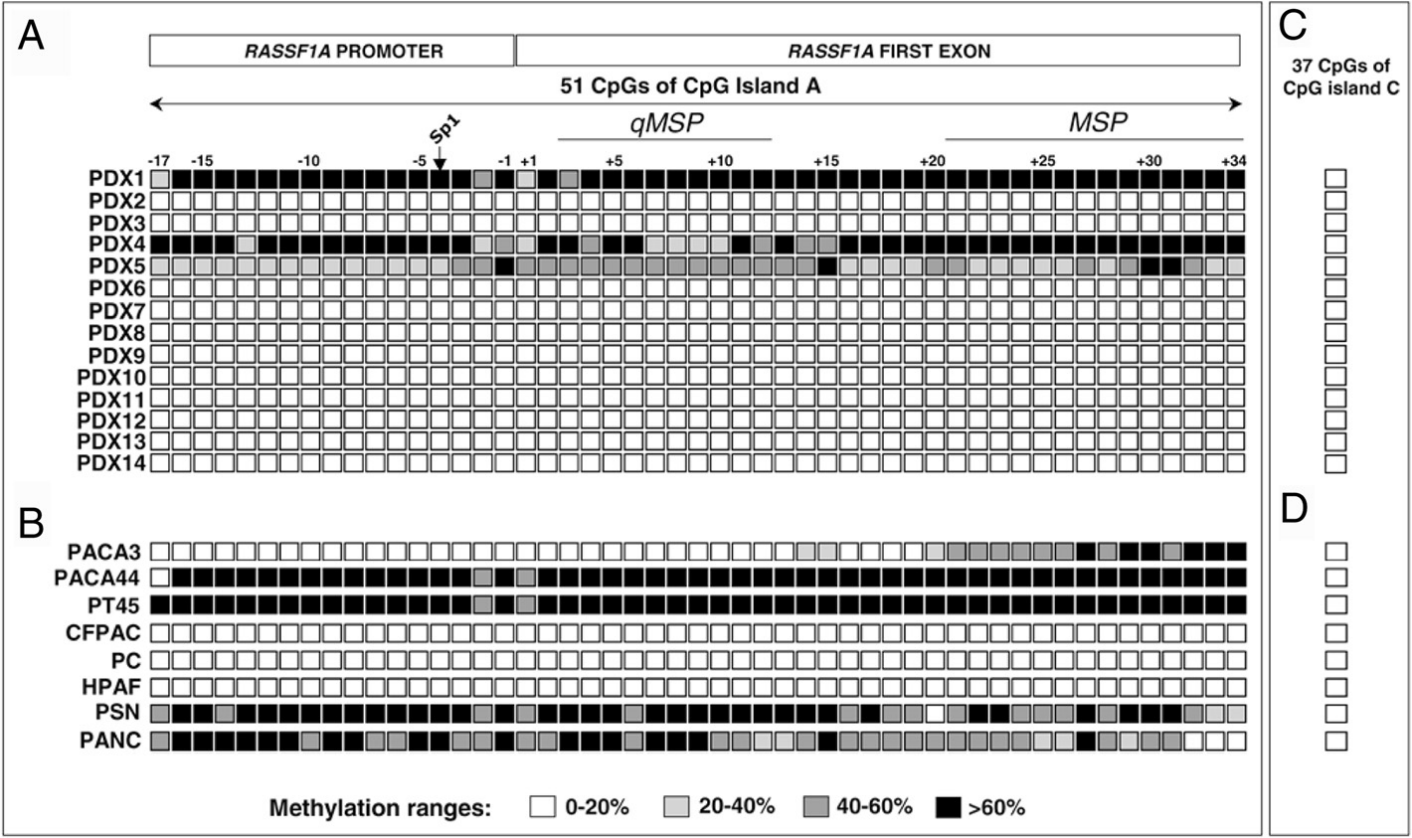
Methylation status of CpG Islands A and C in PDAC xenografts and cell lines. Panel **a** shows the mehylation level of 51 CpGs analyzed by pyrosequencing in 14 PDAC xenografts and eight PDAC cell lines. On the left numbers refer to xenografts and cell lines as listed in Additional file 1: Tables S1 and S2. Each of the 51 CpGs, 17 in the promoter and 34 in the first exon, is represented by a square. Numbers on top show the location of CpG dinucleotides and transcription start site is indicated (+1). Any CpG is represented by a square that has one of four grey levels according to the proportion of methylation detected, namely white, light grey, grey and black indicating a level of methylation of 0–20 %, 20–40 %, 40–60 % and >60 %, respectively. Panel **b** reports the methylation status of the 51 CpGs in the eight indicated PDAC cell lines. Panels **c** and **d** report the methylation status of the 37 CpGs whithin the CpG island C in the indicated PDAC xenografts and cell lines, respectively

DNA pyrosequencing was used to assess the methylation level of 51 CpGs within CpG island A of *RASSF1A* (17 in the promoter and 34 in the first exon) in 14 xenografted primary PDAC and 8 PDAC cell lines. Methylation was detected in 21 % (3/14) of PDAC xenografts (cases PDX1, PDX4 and PDX5) (Fig. 3 and Additional file 1: Table S2) and in 62 % (5/8) of PDAC cell lines (PACA44, PT45, PSN, PANC and PACA3) (Fig. 3 and Additional file 1: Table S3). The average methylation of 51 CpGs was above 70 % in two xenografts (PDX1 and PDX4) and 40 % in one (PDX5). Among the five PDAC cell lines with methylation, two showed an average level higher than 80 % (PACA44, PT45), two between 50 and 70 % (PSN, PANC) and one (PACA3) an average level below 30 %, due to partial methylation of CpGs from CpG14 to CpG34 within the first exon. The methylation status of CpG island A was distributed with no apparent preferential pattern.

None of the 37 CpG of CpG island C (9 in the promoter and 28 in the first exon) investigated by DNA pyrosequencing was methylated in either the 14 PDAC xenografts or the 8 cell lines (Fig. 3).

### The variable expression of RASSF1 isoforms in PDAC xenografts and cell lines is not dependent from CpG islands methylation

The expression levels of the *RASSF1* isoforms were evaluated by real-time qRT-PCR in 14 PDAC xenografts and 8 PDAC cell lines. The three main gene transcripts of the *RASSF1* locus (i.e. RASSF1A, RASSF1B and RASSF1C) were expressed in all xenografts and cell lines (Additional file 2: Figures S2 and S3). RASSF1A and RASSF1B expression levels were correlated (*p* = 0.010, r = 0.8) in both xenografts and cell lines.

Among the remaining 4 mRNA isoforms that are generated from alternative splicing of RASSF1A (RASSF1D, RASSF1E, RASSF1F, RASSF1G), expression of RASSF1F was observed in 8/14 (57 %) PDAC xenografts (PDX1, PDX2, PDX5, PDX6, PDX7, PDX10, PDX12, PDX14) and in 5/8 (62 %) cell lines (PACA3, CFPAC, PC, HPAF, PANC), while isoforms D-E were expressed in 2/14 (14 %) xenografts (PDX7 and PDX14) and in 2/8 (25 %) cell lines (PACA3 and PT45). *RASSF1G* was never expressed in either xenografts or cell lines.

No correlation between the expression level of the *RASSF1* isoforms and methylation of CpG islands was found.

### RASSF1 deregulation is not associated to a specific KRAS mutational profile

Of the 96 presented cases, 92 (96 %) showed mutations in *KRAS* gene and none in *BRAF* gene at Sanger sequencing. Of these 92, we have found: 42 (45.7 %) p.G12D, 31 (33.7 %) p.G12V, 18 (19.5 %) p.G12R, 1 (1.1 %) p.Q61H. There was not a statistical significance between any particular mutation and epigenetic, cytogenetic or clinical parameters. We searched *BRAF* mutations as it has been reported that mutations in this gene may be found in *KRAS* wild-type cancers and rarely may also be associated with *KRAS* mutations [37, 38]. Moreover, BRAF mutations have been reported to be characteristic of the peculiar subtype of pancreas cancer named “medullary” [38, 39], but none of our cases corresponded to such subtype.

### Correlation of expression, methylation and copy number status of RASSF1

*RASSF1* alteration has been suggested as a molecular hallmark of pancreatic cancer [2]. In the attempt to clarify the involvement of *RASSF1* in this setting, we performed a comprehensive analysis of expression, methylation and copy number status of this putative tumor suppressor gene in a relatively large series of primary PDAC, xenografts and PDAC cell lines.

Rassf1a expression was downregulated in about 50 % of samples. However, such downregulation was not correlated with the *RASSF1* methylation status, which indicates that *RASSF1* methylation is not an essential mechanism for regulating protein expression in PDAC. This is the first study investigating RASSF1 isoforms expression in PDAC. RASSF1A, RASSF1B and RASSF1C mRNAs were expressed in all xenografts and cell lines, and, as observed for immunohistochemical data, no correlation between the expression level of the RASSF1 isoforms and methylation of CpG islands was found. However, RASSF1F was preferentially expressed by cell lines lacking methylation at CpG island A (Wilcoxon test, *p* = 0.008), in line with the results of Dammann et al. [2, 3]. This suggests that methylation of CpG island A may affect expression and splicing of RASSF1 transcripts regulated by the same promoter. The lack of correlation between the level of methylation of CpG island A and RASSF1A mRNA level in PDAC xenografts and cell lines further suggests that methylation is not decisive for transcription regulation of RASSF1 isoforms. No correlation was found between methylation and outcome of disease, at variance with other cancer types [13, 16–21]. A consistent number (71/96, 74 %) of *RASSF1* locus alterations was found in our series, including 54 % (50/96) of losses and 22 % (21/96) of gains. The frequency of *RASSF1* loss found is similar to that reported in a previous LOH microsatellite study investigating 82 PDAC xenografts [26]. Notably, among the 50 samples with loss of *RASSF1* locus, 30 (60 %) had a normal chromosome 3 ploidy, while the remaining 20 (40 %) samples had chromosome 3 polysomy. Previous studies documented frequent chromosomal 3 alterations in pancreatic cancer [25–27, 35, 36], including in particular the allelic loss at 3p21.3 [24, 25]. Harada et al. observed frequent gains of chromosome 3 by high-density single nucleotide polymorphism array (78 % of cases), associated with losses in the specific region of chromosome 3p21 [26], this might reflect a propensity for 3p21.3 loss to occur as a secondary event of large 3p deletions, which involves regions coding for other tumor suppressor genes. Interestingly, chromosome 3 poliploidy was associated with a worse prognosis in our series, independently of the status of *RASSF1* locus, while patients with a disomic status of both *CEP3* and *RASSF1* had a similar prognosis compared to patients with disomic *CEP3* and loss of *RASSF1*. It has been described that *RASSF1* methylation can have different roles depending on the ploidy status and patient’s age in neuroblastoma [40]. However, there is no correlation between methylation and ploidy status in our PDAC series. It is possible that other tumor suppressor genes in chromosome 3p may be implicated in the clinical course of these tumors.

## Conclusions

*RASSF1* alterations occurring in PDAC mainly consist in variations of expression of the different isoforms. Different genetic mechanisms seem to contribute to *RASSF1* deregulation in this setting, but *RASSF1* methylation does not seem to substantially affect RASSF1 isoforms expression.

## Additional files

Additional file 1:
**Table S1.** Distribution of Rassf1a immunohistochemical (IHC) expression, *RASSF1* and *CEP3* status analyzed by fluorescence in situ hybridization analysis, and *RASSF1A* methylation-specific PCR (MSP) analysis in 96 PDAC cases. **Table S2.** *RASSF1A* methylation status (DNA pyrosequencing) and Rassf1a immunohistochemical (IHC) expression in 14 xenografted PDAC tissues. Average methylation values of 51 CpGs in CpG island A of *RASSF1A*, of which 17 CpGs in the promoter and 34 CpGs in the first exon are shown. (PDX = PDAC xenografts). **Table S3.** *RASSF1A* methylation status (DNA pyrosequencing) and Rassf1a immunohistochemical (IHC) expression in eight PDAC-derived cell lines. Average methylation values of 51 CpGs in CpG island A of *RASSF1A*, of which 17 CpGs in the promoter and 34 CpGs in the first exon are shown. (DOCX 42 kb) Additional file 2:
**Figure S1.** Survival rate of PDAC patients with *RASSF1* disomy, loss of 3p, loss of 3p with polysomic CEP3 (CEP3+) and excess of CEP3 copy number. **Figure S2.** Expression of RASSF1A, RASSF1B and RASSF1C by quantitative RT-PCR (qRT-PCR) in PDAC xenografts. The expression levels of RASSF1A (**A**), RASSF1B (**B**) and RASSF1C (**C**) in 14 PDAC xenografts are shown. Expression data are the mean of three measures obtained by quantitative real time RT-PCR. Data were normalized using the expression level of the *RPLPO* gene as an internal reference. Data were analyzed according to the comparative method and standard (=1) was represented by the average expression of all samples. Numbers under bars refer to PDAC xenografts (PDX) as listed in Additional file 1: Table S2. **Figure S3.** Expression of RASSF1A, RASSF1B and RASSF1C by quantitative RT-PCR (qRT-PCR) in eight PDAC cell lines. Expression data are the mean of three measures obtained by quantitative real time RT-PCR. Data were normalized using the expression level of the RPLPO gene as an internal reference. Data were analyzed according to the comparative method and standard (=1) was represented by the average expression of all sample. Expression levels (mean + SD) are represented by white bars. (DOC 301 kb)

## Abbreviations

CEP3: centromeric enumeration probe 3
FFPE: formalin-fixed paraffin-embedded
MSP: methylation-specific PCR
PDAC: pancreatic ductal adenocarcinoma
qRT-PCR: quantitative reverse transcription-polymerase chain reaction
RASSF1: ras association domain family member 1
TMA: tissue microarray
