# Supplementary material for: RASSF1 tumor suppressor gene in pancreatic ductal adenocarcinoma: correlation of expression, chromosomal status and epigenetic changes
Source: BMC Cancer. 2016 Jan 12;16:11. doi: 10.1186/s12885-016-2048-0 (PMC4710004; doi:10.1186/s12885-016-2048-0)
Supplement: Additional file 1: — Table S1. Distribution of Rassf1a immunohistochemical (IHC) expression, RASSF1 and CEP3 status analyzed by fluorescence in situ hybridization analysis, and RASSF1A methylation-specific PCR (MSP) analysis in 96 PDAC cases. Table S2. RASSF1A methylation status (DNA pyrosequencing) and Rassf1a immunohistochemical (IHC) expression in 14 xenografted PDAC tissues. Average methylation values of 51 CpGs in CpG island A of RASSF1A, of which 17 CpGs in the promoter and 34 CpGs in the first exon are shown. (PDX = PDAC xenografts). Table S3. RASSF1A methylation status (DNA pyrosequencing) and Rassf1a immunohistochemical (IHC) expression in eight PDAC-derived cell lines. Average methylation values of 51 CpGs in CpG island A of RASSF1A, of which 17 CpGs in the promoter and 34 CpGs in the first exon are shown. (DOCX 42 kb) [file 12885_2016_2048_MOESM1_ESM.docx]

**Additional file 1**

**Table S1**.

| **PDAC** | **Rassf1a**  **IHC** | ***RASSF1* copy number** | ***Chromosome 3 (*CEP3*)* status** | ***RASSF1A* MSP** |
| --- | --- | --- | --- | --- |
| **1** | 0 | Loss | diploid | - |
| **2** | 0 | Loss | diploid | - |
| **3** | 0 | Loss | diploid | - |
| **4** | 0 | Loss | diploid | + |
| **5** | 0 | Loss | diploid | + |
| **6** | 0 | Loss | diploid | + |
| **7** | 1 | Loss | diploid | - |
| **8** | 1 | Loss | diploid | - |
| **9** | 1 | Loss | diploid | - |
| **10** | 1 | Loss | diploid | + |
| **11** | 1 | Loss | diploid | - |
| **12** | 1 | Loss | diploid | - |
| **13** | 1 | Loss | diploid | + |
| **14** | 1 | Loss | diploid | - |
| **15** | 1 | Loss | diploid | - |
| **16** | 1 | Loss | diploid | - |
| **17** | 1 | Loss | diploid | + |
| **18** | 1 | Loss | diploid | - |
| **19** | 1 | Loss | diploid | + |
| **20** | 1 | Loss | diploid | - |
| **21** | 1 | Loss | diploid | + |
| **22** | 2 | Loss | diploid | - |
| **23** | 2 | Loss | diploid | - |
| **24** | 2 | Loss | diploid | + |
| **25** | 2 | Loss | diploid | - |
| **26** | 2 | Loss | diploid | - |
| **27** | 2 | Loss | diploid | + |
| **28** | 2 | Loss | diploid | + |
| **29** | 2 | Loss | diploid | - |
| **30** | 3 | Loss | diploid | - |
| **31** | 0 | no loss | diploid | - |
| **32** | 1 | no loss | diploid | - |
| **33** | 1 | no loss | diploid | - |
| **34** | 1 | no loss | diploid | - |
| **35** | 1 | no loss | diploid | + |
| **36** | 1 | no loss | diploid | - |
| **37** | 1 | no loss | diploid | - |
| **38** | 1 | no loss | diploid | + |
| **39** | 1 | no loss | diploid | - |
| **40** | 1 | no loss | diploid | + |
| **41** | 1 | no loss | diploid | - |
| **42** | 1 | no loss | diploid | - |
| **43** | 1 | no loss | diploid | + |
| **44** | 2 | no loss | diploid | - |
| **45** | 2 | no loss | diploid | + |
| **46** | 2 | no loss | diploid | + |
| **47** | 2 | no loss | diploid | - |
| **48** | 2 | no loss | diploid | + |
| **49** | 2 | no loss | diploid | - |
| **50** | 2 | no loss | diploid | - |
| **51** | 2 | no loss | diploid | + |
| **52** | 3 | no loss | diploid | - |
| **53** | 3 | no loss | diploid | - |
| **54** | 3 | no loss | diploid | - |
| **55** | 3 | no loss | diploid | - |
| **56** | 1 | Loss | polyploid | - |
| **57** | 1 | Loss | polyploid | + |
| **58** | 1 | Loss | polyploid | - |
| **59** | 1 | Loss | polyploid | - |
| **60** | 1 | Loss | polyploid | - |
| **61** | 1 | Loss | polyploid | + |
| **62** | 1 | Loss | polyploid | - |
| **63** | 1 | Loss | polyploid | + |
| **64** | 2 | Loss | polyploid | - |
| **65** | 2 | Loss | polyploid | - |
| **66** | 2 | Loss | polyploid | + |
| **67** | 2 | Loss | polyploid | - |
| **68** | 2 | Loss | polyploid | - |
| **69** | 2 | Loss | polyploid | - |
| **70** | 2 | Loss | polyploid | + |
| **71** | 2 | Loss | polyploid | + |
| **72** | 2 | Loss | polyploid | + |
| **73** | 3 | Loss | polyploid | - |
| **74** | 3 | Loss | polyploid | + |
| **75** | 3 | Loss | polyploid | - |
| **76** | 1 | no loss | polyploid | - |
| **77** | 1 | no loss | polyploid | - |
| **78** | 1 | no loss | polyploid | - |
| **79** | 1 | no loss | polyploid | - |
| **80** | 1 | no loss | polyploid | - |
| **81** | 1 | no loss | polyploid | + |
| **82** | 2 | no loss | polyploid | - |
| **83** | 2 | no loss | polyploid | + |
| **84** | 2 | no loss | polyploid | - |
| **85** | 2 | no loss | polyploid | - |
| **86** | 2 | no loss | polyploid | - |
| **87** | 2 | no loss | polyploid | + |
| **88** | 2 | no loss | polyploid | - |
| **89** | 2 | no loss | polyploid | + |
| **90** | 2 | no loss | polyploid | - |
| **91** | 2 | no loss | polyploid | + |
| **92** | 3 | no loss | polyploid | - |
| **93** | 3 | no loss | polyploid | + |
| **94** | 3 | no loss | polyploid | - |
| **95** | 3 | no loss | polyploid | - |
| **96** | 3 | no loss | polyploid | + |
| CEP3, centromeric enumeration probe of chromosome 3; -, MSP negative; +, MSP positive. | | | | |

**Table S2.**

| **Cases** | **Average methylation *RASSF1A* (%)** | | **Rassf1A IHC** |
| --- | --- | --- | --- |
|  | **Promoter** | **First Exon** | **Score** |
| PDX1 | 88 | 87 | 1+ |
| PDX2 | 12 | 2 | 0 |
| PDX3 | 6 | 8 | 0 |
| PDX4 | 82 | 74 | 1+ |
| PDX5 | 38 | 41 | 2+ |
| PDX6 | 3 | 3 | 2+ |
| PDX7 | 3 | 2 | 1+ |
| PDX8 | 2 | 1 | 1+ |
| PDX9 | 3 | 2 | 2+ |
| PDX10 | 4 | 4 | 1+ |
| PDX11 | 3 | 2 | 2+ |
| PDX12 | 5 | 3 | 2+ |
| PDX13 | 4 | 2 | 0 |
| PDX14 | 3 | 3 | 2+ |

**Table S3.**

| **Cell lines** | **Averagemethylation *RASSF1A*(%)** | | **Rassf1A IHC** |
| --- | --- | --- | --- |
|  | **Promoter** | **First Exon** | **Score** |
| PACA3 | 10 | 33 | 2+ |
| PACA44 | 79 | 85 | 1+ |
| PT45 | 86 | 86 | 1+ |
| CFPAC | 5 | 5 | 2+ |
| PC | 5 | 3 | 1+ |
| HPAF | 7 | 5 | 2+ |
| PSN | 70 | 62 | 1+ |
| PANC | 67 | 51 | 3+ |
