# Supplementary figures and images for: RASSF1 tumor suppressor gene in pancreatic ductal adenocarcinoma: correlation of expression, chromosomal status and epigenetic changes
Source: BMC Cancer. 2016 Jan 12;16:11. doi: 10.1186/s12885-016-2048-0 (PMC4710004; doi:10.1186/s12885-016-2048-0)

**Additional file 2**

**Figure S1.**


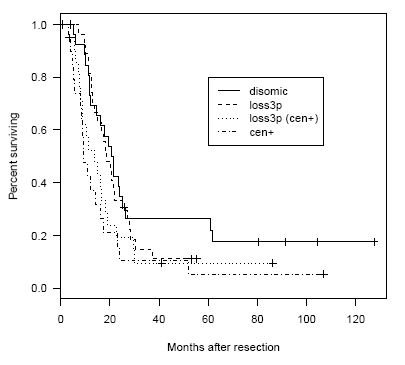


**Figure S2.**


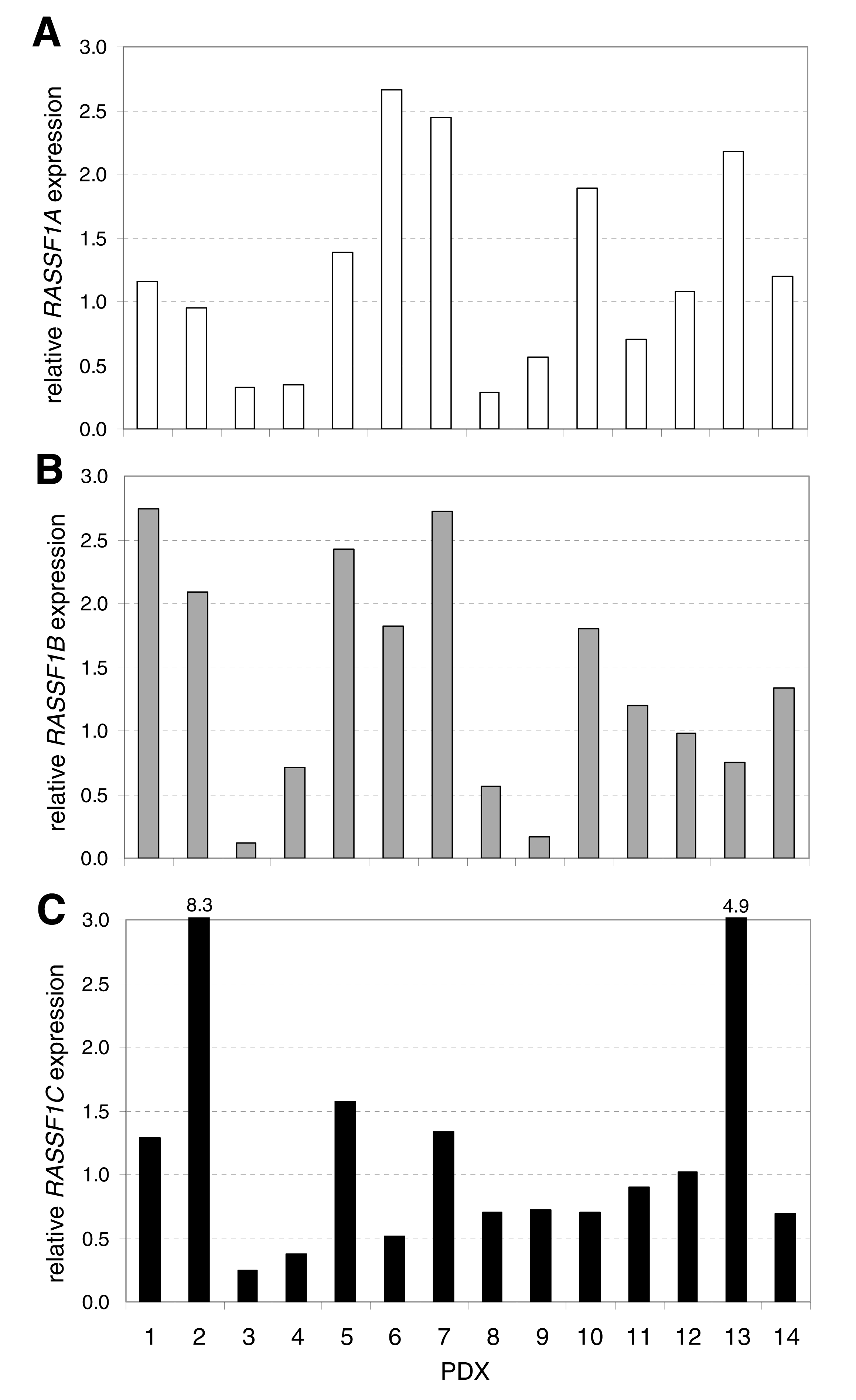


**Figure S3.**

**
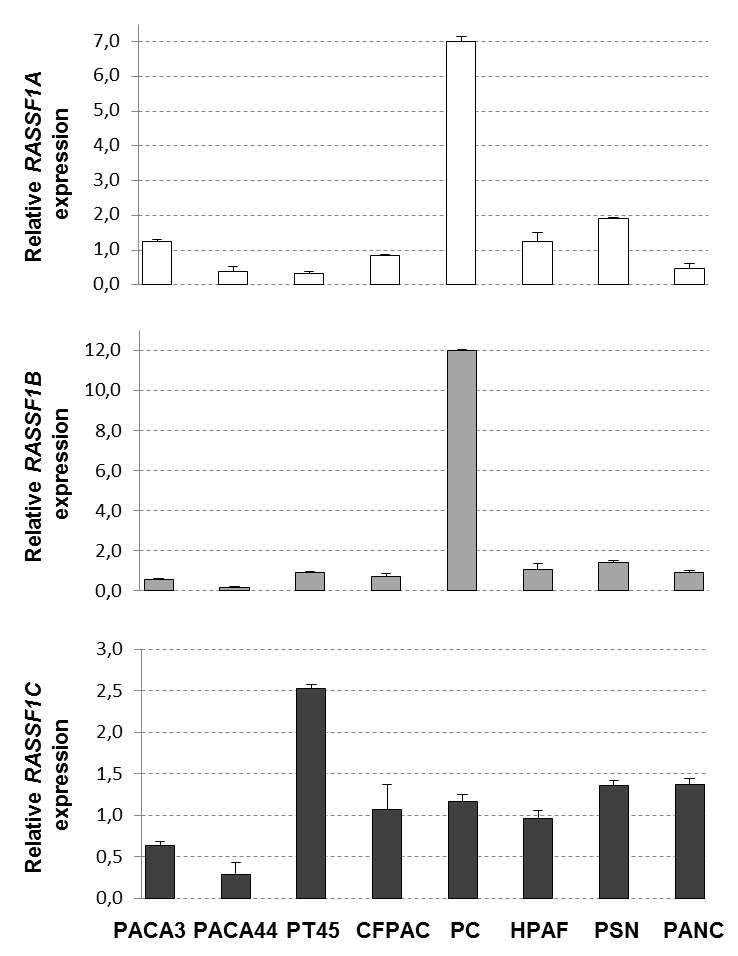
**

Supplement: Additional file 2: — Figure S1. Survival rate of PDAC patients with RASSF1 disomy, loss of 3p, loss of 3p with polysomic CEP3 (CEP3+) and excess of CEP3 copy number. Figure S2. Expression of RASSF1A, RASSF1B and RASSF1C by quantitative RT-PCR (qRT-PCR) in PDAC xenografts. The expression levels of RASSF1A (A), RASSF1B (B) and RASSF1C (C) in 14 PDAC xenografts are shown. Expression data are the mean of three measures obtained by quantitative real time RT-PCR. Data were normalized using the expression level of the RPLPO gene as an internal reference. Data were analyzed according to the comparative method and standard (=1) was represented by the average expression of all samples. Numbers under bars refer to PDAC xenografts (PDX) as listed in Additional file 1: Table S2. Figure S3. Expression of RASSF1A, RASSF1B and RASSF1C by quantitative RT-PCR (qRT-PCR) in eight PDAC cell lines. Expression data are the mean of three measures obtained by quantitative real time RT-PCR. Data were normalized using the expression level of the RPLPO gene as an internal reference. Data were analyzed according to the comparative method and standard (=1) was represented by the average expression of all sample. Expression levels (mean + SD) are represented by white bars. (DOC 301 kb) [file 12885_2016_2048_MOESM2_ESM.doc]
